# A concentrated machine learning-based classification system for age-related macular degeneration (AMD) diagnosis using fundus images

**DOI:** 10.1038/s41598-024-52131-2

**Published:** 2024-01-29

**Authors:** Aya A. Abd El-Khalek, Hossam Magdy Balaha, Norah Saleh Alghamdi, Mohammed Ghazal, Abeer T. Khalil, Mohy Eldin A. Abo-Elsoud, Ayman El-Baz

**Affiliations:** 1 Communications and Electronics Engineering Department, Nile Higher Institute for Engineering and Technology, Mansoura, Egypt; 2 BioImaging Lab, Department of Bioengineering, J.B. Speed School of Engineering, University of Louisville, Louisville, KY USA; 3 Department of Computer Sciences, College of Computer and Information Sciences, Princess Nourah bint Abdulrahman University, Riyadh, Saudi Arabia; 4 Electrical, Computer, and Biomedical Engineering Depatrment, Abu Dhabi University, Abu Dhabi, UAE; 5 Communications and Electronics Engineering Department, Faculty of Engineering, Mansoura University, Mansoura, Egypt

**Keywords:** Biological techniques, Biomarkers

## Abstract

The increase in eye disorders among older individuals has raised concerns, necessitating early detection through regular eye examinations. Age-related macular degeneration (AMD), a prevalent condition in individuals over 45, is a leading cause of vision impairment in the elderly. This paper presents a comprehensive computer-aided diagnosis (CAD) framework to categorize fundus images into geographic atrophy (GA), intermediate AMD, normal, and wet AMD categories. This is crucial for early detection and precise diagnosis of age-related macular degeneration (AMD), enabling timely intervention and personalized treatment strategies. We have developed a novel system that extracts both local and global appearance markers from fundus images. These markers are obtained from the entire retina and iso-regions aligned with the optical disc. Applying weighted majority voting on the best classifiers improves performance, resulting in an accuracy of 96.85%, sensitivity of 93.72%, specificity of 97.89%, precision of 93.86%, F1 of 93.72%, ROC of 95.85%, balanced accuracy of 95.81%, and weighted sum of 95.38%. This system not only achieves high accuracy but also provides a detailed assessment of the severity of each retinal region. This approach ensures that the final diagnosis aligns with the physician’s understanding of AMD, aiding them in ongoing treatment and follow-up for AMD patients.

## Introduction

Eye disorders have become a growing concern among older individuals in recent years. Often, these conditions progress unnoticed until symptoms appear, emphasizing the importance of regular eye examinations for early detection^1^. This is especially critical when it comes to Age-related Macular Degeneration (AMD), a prevalent condition that affects individuals over 45 and is one of the leading causes of vision impairment in the elderly^2^. Traditional diagnosis methods like slit-lamp examinations by ophthalmologists have limitations due to skill variations and record-keeping issues. However, there is a promising avenue for AMD diagnosis and management through the use of machine learning (ML) algorithms for classifying fundus images^3^.

Located on the outer pole of the retina, the macula plays an important role in sharp color vision. Abnormalities in this region can cause blurred vision, dark circles, and malformations. The exact causes of AMD are not well understood, but genetics, chronic light exposure and nutritional imbalances are involved. To better understand AMD progression and effective treatment, it is necessary to classify fundus images into groups such as geographic atrophy (GA), intermediate AMD, normal, wet AMD, etc.^4,5^. GA stands for dry advanced stage with progressive retinal pigment epithelium (RPE) cell loss, resulting in distinct atrophic patches and central vision loss. Intermediate AMD falls between early and advanced stages, characterized by drusen pigment changes. The “normal” category includes images without AMD-related changes, which generally do not show clinical evidence of the disease. Wet AMD, the severe form, involves abnormal blood vessel growth beneath the retina, leading to retinal degeneration and, if left untreated, rapid loss of central vision^6–8^.

ML techniques hold significant promise in precisely categorizing fundus images into different AMD stages. These methods efficiently analyze extensive datasets and acquire intricate patterns and relationships from images, allowing for the identification of subtle indicators of various AMD phases. By automating the classification process, ML algorithms offer consistent and objective assessments, reducing discrepancies between observers and facilitating prompt diagnoses. A range of ML algorithms have been explored for the classification of AMD fundus images, as documented in^9^.

Traditional ML methods^10^ such as random forest (RF), multilevel perceptron (MLP), decision tree (DT), logistic regression (LR), support vector machines (SVM), and K-nearest neighbors (KNN) have shown promising results in previous studies, showing exceptional performance in image classification tasks^11^. Reliable ML-based settings for classifying fundus images into GA, intermediate AMD, normal, wet AMD categories have great potential for clinical application. Such systems can help eyesight specialists to provide accurate and timely diagnosis, facilitating appropriate treatment options for patients^12^. Furthermore, it can play an important role in large-scale screening programs, enhancing early detection and intervention, and ultimately improving the visual outcome of individuals with AMD^13^.

The aim of the present study is to evaluate and investigate the accuracy of ML methods for classifying AMD stages from fundus images. Aside from revealing the entire selection process, the analysis also considers the metrics employed to examine the developed classification model. The paper further explores the findings, possible obstacles and future approaches to make the ML-based AMD classification systems more accurate and clinically useful. In this regard, this review advances knowledge in this domain and paves the way for better patient care and outcomes associated with computer-aided AMD. The following points provide a summary of the present study’s contributions:

- Development of a non-invasive CAD system: the study successfully developed a non-invasive CAD system for AMD using ML methods, which provides a valuable tool early diagnosis of his disease.
- Improved AMD classification: through extensive research and testing, the study enhanced the accuracy and reliability of AMD classification from fundus images. This improvement ensures a more equitable classification of AMD within different stages.
- Enhanced patient care: the CAD system, by automating the AMD diagnostic process and bridging gaps between caregivers, has the potential to significantly improve patient care. This advancement ensures timely and accurate inspections, contributing to enhanced overall healthcare delivery.

### Paper organization

The paper is organized as follows: “Related studies” discusses related work for AMD classification. “Materials” describes the research materials. “Methodology” presents the proposed approach for AMD classification and its phases in details. “Experiments” presents the experimental result and discussion. “Overall discussion” discusses the work and experiments. “Limitations” highlight the study’s limitations. Finally, “Conclusions and future directions” addresses the conclusions and future directions.

## Related studies

Recently, several algorithms have been developed to address the challenge of classifying fundus images of age-related macular degeneration (AMD) by leveraging patterns and features present in the data. These efforts have resulted in a significant body of academic work focused on the classification of AMD fundus images. Furthermore, various classification techniques and methodologies have been explored in these studies.

Notable examples include the work of Bhuiyan et al.^14^, who used convolutional neural networks (CNNs) to classify Referable AMD using the AREDS dataset which contains about 116,875 images. The results show that the classification for Disease/no disease provides better results with about 0.992 accuracy and for AMD severity (4 classes) with about 0.961 accuracy. Zapata et al.^15^ Proposed a classification approach using CNNs for AMD Disease/no disease using the Optretina dataset which contains about 306,302 images. This research achieved an accuracy of 0.863 and an AUC of 0.936.

Bulut et al.^16^ proposed a deep learning approach (i.e., Xception model) for detecting retinal abnormalities based on color fundus images. During the analysis, the Xception model containing 50 different parameter combinations was trained. The highest accuracy achieved was 82.5%. Gayathri et al.^17^ proposed an automated binary and multiclass classification of diabetic retinopathy. The proposed work focuses on the extraction of Haralick and Anisotropic Dual-Tree Complex Wavelet Transform (ADTCWT) features that can perform reliable DR classification from retinal fundus images. The evaluation results show that by applying the proposed feature extraction method, Random Forest outperforms all the other classifiers with an average accuracy of 99.7% and 99.82% for binary and multiclass classification, respectively.

Furthermore, Rajagopalan et al.^18^ proposed a deep convolution neural network (DCNN) architecture for the classification and diagnosis of average diabetic macular edema (DME) and drusen macular degeneration (DMD) efficiently. Firstly, the despeckling of the input OCT image is executed by the Kuan filters to remove inherent speckle noise. Furthermore, the CNN networks are tuned with hyper-parameter optimization methods. Moreover, K-fold validations are performed to guarantee full use of the datasets. Chakravorti et al.^19^ proposed an efficient CNN for AMD classification. The network was trained on fundus images to classify them into the four AMD categories, achieving high accuracy with reduced computational complexity. Thomas et al.^20^ developed an algorithm for the diagnosis of AMD in retinal OCT images based on the detection of RPE layers and the baseline estimate of statistical approaches and randomization.

Additionally, Zheng et al.^21^ designed a five-category intelligent auxiliary diagnosis model for common fundus diseases. The accuracy rates of the 3 intelligent auxiliary diagnosis models were all above 90%, and the kappa values were all above 88%. For the 4 common fundus diseases, the best results of sensitivity, specificity, and F1-scores were 97.12%, 99.52%, 96.43%, and 98.21%, respectively. Vaiyapuri et al.^22^ presented a new multi-retinal disease diagnosis model using the IDL-MRDD technique to determine different types of retinal diseases. The experimental values pointed out the superior outcome over the existing techniques with a maximum accuracy of 0.963. Lee et al.^23^ proposed two deep learning models, CNN-LSTM and CNN-Transformer, which use a Long-Short Term Memory (LSTM) and a Transformer, respectively with CNN, to capture the sequential information in longitudinal CFPs. The proposed models outperformed the baseline models that utilized only single-visit CFPs to predict the risk of late AMD (0.879 vs. 0.868 in AUC for 2-year prediction, and 0.879 vs. 0.862 for 5-year prediction).

Moreover, Kar et al.^24^ introduced an innovative method for precise retinal blood vessel detection in fundus images. Their approach features a generative adversarial network (GAN)^25^ with a unique architecture, combining a multi-scale residual convolutional neural network as the generator and a vision transformer as the discriminator. The GAN model, employing adversarial learning, achieves state-of-the-art results. Preprocessing involves contrast enhancement using a contrast-limited adaptive histogram equalization algorithm. Rigorous evaluations on multiple databases confirm the method’s robustness and efficacy, outperforming existing approaches with notable accuracy scores on CHASE_DB1, DRIVE, HRF, and ARIA databases.

In addition, Elangovan et al.^26^ proposed a robust automated glaucoma diagnosis system utilizing a deep ensemble model and stacking ensemble learning. The study focuses on the efficiency of thirteen pre-trained models, including Alexnet, Googlenet, VGG-16, VGG-19, Squeezenet, Resnet-18, Resnet-50, Resnet-101, Efficientnet-b0, Mobilenet-v2, Densenet-201, Inception-v3, and Xception. The ensemble model, evaluated in 65 configurations, employs a two-stage ensemble selection technique and a probability averaging approach. The final classification integrates an SVM classifier. The method demonstrates exceptional performance on modified publicly available databases (DRISHTI-GS1-R, ORIGA-R, RIM-ONE2-R, LAG-R, and ACRIMA-R), achieving overall classification accuracies of 93.4%, 79.6%, 91.3%, 99.5%, and 99.6%, respectively.

Furthermore, Haider et al.^27^ introduced ESS-Net and FBSS-Net for accurate OD and OC segmentation in retinal fundus images, addressing challenges like size and pixel variations. Both networks, with 3.02 million trainable parameters, demonstrated excellent segmentation on datasets like REFUGE and Drishti-GS, providing efficient solutions for computer-assisted glaucoma diagnosis. Additionally, Arsalan et al.^28^ introduced the vessel segmentation ultra-lite network (VSUL-Net) to accurately extract retinal vasculature without image preprocessing. With only 0.37 million trainable parameters, VSUL-Net utilizes a retention block for improved sensitivity, eliminating the need for expensive preprocessing schemes. Tested on DRIVE, STARE, and CHASE-DB1 datasets, the method achieved robust segmentation with Sensitivity, Specificity, Accuracy, and Area Under the Curve values of 83.80%, 98.21%, 96.95%, and 98.54% for DRIVE, 81.73%, 98.35%, 97.17%, and 98.69% for CHASE-DB1, and 86.64%, 98.13%, 97.27%, and 99.01% for STARE datasets.

Similarly, Singh et al.^29^ proposed an efficient glaucoma detection system using customized particle swarm optimization (CPSO) and four state-of-the-art machine-learning classifiers. The interconnected architecture involves pre-processing, segmentation, feature extraction, selection of critical features, and classification using CPSO-machine learning. The study focuses on a public dataset, Digital Retinal Images for Optic Nerve Segmentation. Unlike using all 20 extracted features, the system selects critical features based on univariate and feature importance methods. The best performance is achieved with a CPSO-K-nearest neighbor hybrid method, recording a maximum accuracy of 0.99, specificity of 0.96, sensitivity of 0.97, precision of 0.97, F1-score of 0.97, and Kappa of 0.94. Singh et al.^30,31^ also addressed feature selection challenges in machine learning, focusing on glaucoma detection using benchmark datasets. The study introduces a metaheuristics-based feature selection technique employing emperor penguin optimization and bacterial foraging optimization, proposing a hybrid algorithm. From 36 features extracted from retinal fundus images, the technique minimizes the feature set while enhancing classification accuracy. Six machine learning classifiers evaluate smaller subsets provided by the optimization techniques. The hybrid optimization technique, paired with random forest, achieves the highest accuracy at 0.95410.

In summary, these studies collectively provide valuable insights into the performance of diverse classification techniques for AMD fundus image classification. The results highlight the effectiveness of deep learning methods and the importance of feature extraction techniques in achieving accurate and reliable classifications. While Random Forest and SVM often excel in terms of classification accuracy, it is crucial to consider the dataset, feature extraction methods, and evaluation metrics when interpreting specific results from these studies.

While previous investigations have provided valuable insights into the performance of diverse classification techniques for AMD fundus image classification, there remains a notable gap in the interpretation of the data by extracting both local and global features from it. Many of the mentioned studies have focused on the application of deep learning and convolutional neural networks (CNNs) for classification, achieving impressive accuracy rates. However, these studies have predominantly emphasized the utilization of deep learning methods without comprehensive exploration of feature extraction techniques that capture both local and global characteristics of fundus images.

The extraction of local features, which pertain to specific regions or structures within the image, can provide valuable information about subtle abnormalities in the retina. Similarly, global features, which encompass broader characteristics of the entire image, can offer insights into overall patterns and structures. Combining both types of features can enhance the interpretability of the classification process and potentially lead to more robust and explainable results.

## Materials

### Patient selection and characteristics

Patient selection required the collection of retinal fundus images from a diverse group of real patients who showed symptoms associated with AMD, including different stages and types of disease. The database used in this study consists of more than 864 retinal images, including AMD. Each patient’s demographic and clinical characteristics, including age, sex, and their specific AMD category, were recorded. The experimental protocols were approved by the authors’ and patients’ institutions: University of Louisville and Mansoura University.

### Imaging techniques

The retinal imaging techniques used in this study primarily used fundus color imaging. These two-dimensional images were obtained by light-reflecting retina^32^. Complete data were obtained by performing the left and right eyes of each patient. Using state-of-the-art imaging equipment, high-quality, and high-resolution images were obtained.

- Fundus color images were taken with standard retinal imaging techniques.
- High quality/resolution imaging equipment was used to ensure image quality and detail.
- Images of the left and right eyes were obtained for each patient for all analyses.

### Data collection and analysis

Data collection involved the systematic acquisition of retinal images from the fundus images maintained for this study. The collected data were intensively analyzed and subsequently extracted relevant features and attributes necessary for an ML-based diagnostic model.

### Data categorization

The data used in this study included four distinct types of AMD, namely geographic atrophy (GA), central, normal AMD, and wet AMD. Each category represents a different stage or form of AMD. Classification of cases was based on clinical evaluation and expert review, which ensured classification accuracy.

### Study design and ethical considerations

In the context of this research, a study design was established to investigate the use of ML-based medical diagnostic techniques for the classification of AMD using retinal fundus images. Ethical considerations were taken into account, and all procedures adhered to the relevant ethical guidelines and regulations. Informed consent was obtained from all patients involved in the study. The current study does not contain any studies with human participants and/or animals performed by any of the authors.

### Consent to participate

All patients have provided informed consent for the present study.

## Methodology

**Figure 1:**
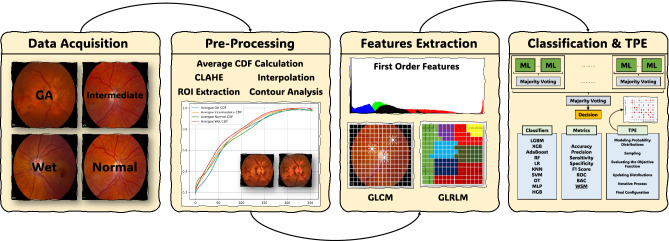
The proposed framework for AMD diagnosis in the current study comprises distinct stages, including data acquisition, pre-processing, feature extraction, classification, and the utilization of Tree of Parzen Estimators (TPE) for optimization.

A detailed CAD framework for AMD analysis is introduced and shown graphically in Fig. 1. This involves the procedures involved in the data acquisition to obtain the necessary information. Pre-processing techniques are then applied to improve subsequent data quality. The extraction methods used to extract meaningful patterns from the data. The classification stage helps in an accurate classification of AMD cases. Moreover, Tree of Parzen Estimators (TPE) is used as another tool to enhance the models. This method is reliable and guarantees a precise diagnosis of AMD; thus, it offers a viable strategy for improving people’s health.

### Data pre-processing phase

**Figure 2:**
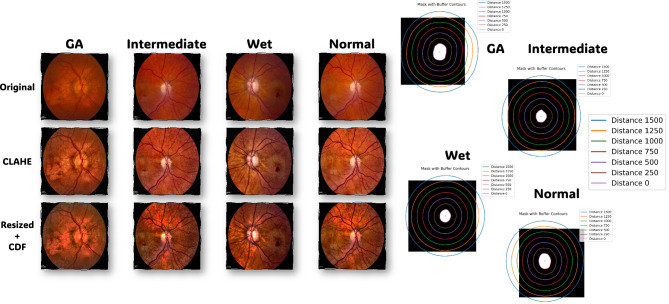
Image pre-processing steps: illustration of the various stages of image preparation for dataset samples. The top row displays the original data. The middle row shows the data after applying CLAHE. The bottom row exhibits the resized data following CDF interpolation. On the right side, you can see contours at different distances from 0 (representing the original mask) up to 1500.

Data preprocessing is the important stage in image analysis consisting of some steps which aim at improving data quality and its preparation for further analysis^33,34^. The data preprocessing pipeline (Fig. 2) denotes a systematic way of preparing a dataset for analysis with an appropriate basis as outlined above. The suggested pipeline is split into a set of step where each is designed to enhance the upcoming classification quality and consistency:

- Average CDF calculation: calculate the average CDF for the different classes to understand the pixel intensity distribution.
- CLAHE enhancements: apply the CLAHE algorithm to improve image contrast and reducing noise.
- Interpolation: interpolate the individual image CDFs with the mean CDF to standardize the intensity distribution.
- ROI extraction: use masks to extract regions of interest from images, focusing on relevant regions.
- Contour analysis: use contour analysis to adjust ROIs, define object boundaries, and calculate object properties.

### Average Cumulative Distribution Function for Class-Specific Pixel Intensities

**Figure 3:**
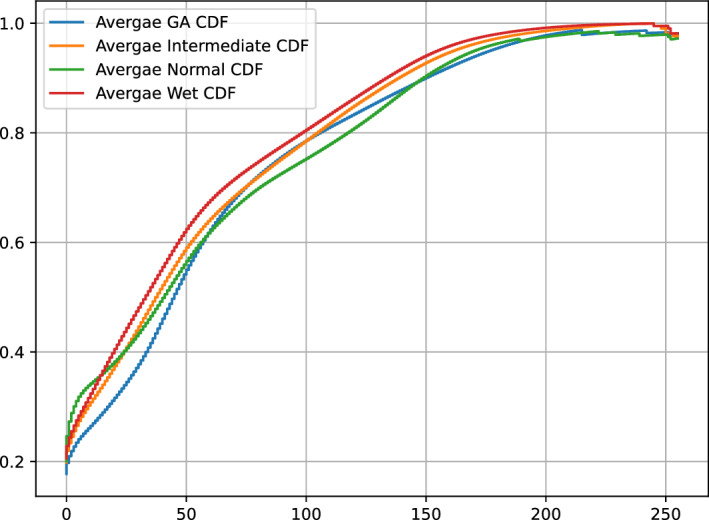
Visualization of the normalized average CDFs for each class: GA, Intermediate, Normal, and Wet. The x-axis is the intensity while the y-axis is the probability.

An important concept in data preprocessing is the cumulative distribution function (CDF). An CDF represents the cumulative probability distribution of pixel intensities in an image, and provides valuable insight into its properties. To prepare our data, we calculate the average CDF (ACDF) for each class within our dataset. This average CDF, denoted as $ACDF_C(x)$ for class *C*, is computed by aggregating the individual CDFs of all images in that class. The mathematical representation is as follows: $ACDF_C(x) = \frac{1}{N_C} \times \sum _{i=0}^{N_C}{{CDF_{C_{i}}(x)}}$ where, $ACDF_C(x)$ represents the average CDF for class *C* at pixel intensity *x*, $N_C$ is the total number of images in class *C*, and ${{CDF_{C_{i}}(x)}}$ is the CDF of the i-th image in class *C*. Figure 3 shows the normalized average CDFS for each class.

### Contrast limited adaptive histogram equalization (CLAHE)

Histogram equalization is a technique used to enhance image contrast by redistributing pixel intensities. However, it can inadvertently amplify noise^35^. Contrast Limited Adaptive Histogram Equalization (CLAHE) builds on this concept by applying histogram equalization locally, in small regions of an image. The theoretical foundation of CLAHE involves several key aspects:

- Histogram equalization: traditional histogram equalization stretches the intensity values across the entire image, aiming for a uniform distribution. Mathematically, it can be represented as: $CLAHE(x,y)=CDF_{clip}(I(x,y))$ where *CLAHE*(*x*, *y*) denotes the CLAHE-enhanced pixel intensity at coordinates (*x*, *y*), and $CDF_{clip}(I(x,y))$ is the clipped CDF of the pixel intensity *I*(*x*, *y*).
- Adaptive approach: CLAHE adapts the histogram equalization process by dividing the image into smaller tiles or regions. Each region is equalized independently, allowing for localized contrast enhancement.
- Contrast limiting: to prevent excessive amplification of pixel values, CLAHE limits the slope of the CDF within each region. This limitation balances contrast enhancement with noise control.

### Interpolate the average CDF with images

Interpolation is a vital step in making the individual images to conform to the average CDF of their classes. This guarantees that images in a given class are uniform in terms of intensity distribution and this, therefore, ensures ease of comparisons and later analysis. The interpolation operation is defined as follows: $I_{eq}(x,y) = InterpolateCDF(I(x,y), targetFreq, targetBins)$ where $I_{eq}(x,y)$ represents the equalized pixel intensity at coordinates (*x*, *y*), and *InterpolateCDF*(*I*(*x*, *y*), *targetFreq*, *targetBins*) is the interpolation function. The goal is to adjust pixel values of each image so that they align with the target CDF, effectively normalizing the data.

### Extract the ROIs using masks

The ROIs are important to the analysis, since they represent parts of images that contain important information^36,37^. This is achieved by means of binary masks, where values of 1 represent the region of interest and 0 indicates background. The image is multiplied with a binary mask, that is, the pixel-wise product, where the areas of interest are selected while the background is set to zero.

### Contours handling

**Figure 4:**
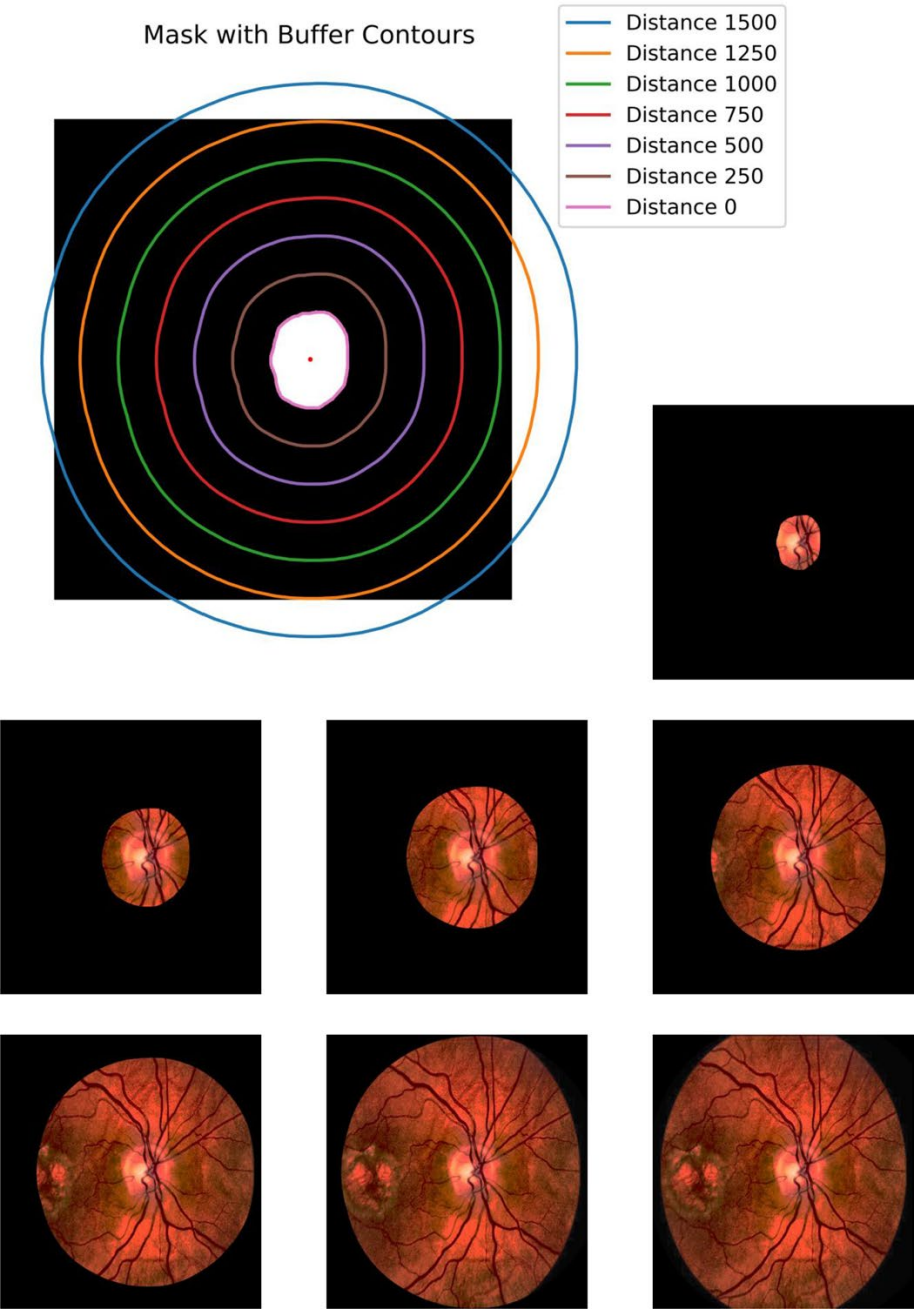
Contours handling: visualization of the extracted ROIs on a sample. It shows the the ROIS at distances from 0 (representing the original mask) up to 1500.

Contour analysis is a pivotal step in refining ROIs and identifying object boundaries within images^38,39^. Contours represent the boundaries of objects and offer valuable properties like area, perimeter, and centroid^40^. The centroid $(C_x,C_y)$ of an object within a contour is calculated using moments, which are mathematical descriptors of the shape and spatial distribution of an object. Moments are defined as: $C_x=\frac{M_{10}}{M00}$ and $C_y=\frac{M_{01}}{M_{00}}$ where $C_x$ and $C_y$ are the *x* and *y* coordinates of the centroid, respectively, and $M_{00}$ is the zeroth moment (i.e., total area of the contour). Contour analysis allows us to precisely locate object boundaries, measure object properties, and define buffer regions, essential for various image analysis tasks. The contours are take at different distances from 0 (representing the original mask) up to 1500. Figure 4 represents a visualization of the extracted ROIs on a sample.

### Features extraction phase

In the field of image processing and texture analysis, feature extraction plays a crucial role in quantifying the characteristics of an image. The study worked on extracting both first-order and second-order features using GLCM (Gray-Level Co-occurrence Matrix) and GLRLM (Gray-Level Run-Length Matrix) methods for each extracted contour resulted from the pre-processing step^41^.

GLCM is a statistical method used to capture the spatial relationships between pixel values in an image. It is defined based on the co-occurrence of pairs of pixel values at various distances and angles in the image^42^. GLCM is typically calculated for a given gray-level image I, with discrete gray levels $\{0, 1,\ldots , L-1\}$. Second-order texture features consider the spatial relationships between pixel pairs in an image. These features provide information about how pixel intensities are distributed relative to each other. Equations of the first- and second-order features were presented in the appendix.

### Scaling phase

The current study utilized several data scaling techniques to preprocess the dataset effectively, enhancing the performance of ML models. These scaling methods are crucial for ensuring that features are on compatible scales and optimizing the behavior of various algorithms during the analysis^43,44^. The study employed four main scaling techniques: Standardization (Z-score Scaling), Min-Max Scaling (Normalization), and Max Absolute Scaling.

Standardization (Eq. 1) transforms data to have a mean of 0 and a standard deviation of 1. It is beneficial when dealing with features of different units, making them comparable by subtracting the mean and dividing by the standard deviation^45^. Min-Max scaling (Eq. 2) scales data to a specified range, often between 0 and 1. It maintains relative relationships between data points by subtracting the minimum value and dividing by the range^46^. Max Absolute (Eq. 3) scaling scales data based on the maximum absolute value within each feature, maintaining the sign of the data while restricting it within a consistent range^47^.

1 $$\begin{aligned} X_{i_{new}}= & {} \frac{X_i-\mu _i}{\sigma _i}. \end{aligned}$$

2 $$\begin{aligned} X_{i_{new}}= & {} \frac{X_{i}-X_{i_{min}}}{X_{i_{max}}-X_{i_{min}}}. \end{aligned}$$

3 $$\begin{aligned} X_{i_{new}}= & {} \frac{X_{i}}{\max (|X_{i}|)}. \end{aligned}$$

### Classification and optimization phase

The present study used advanced classification schemes to locate and analyze the data. These algorithms include various methods, such as LightGBM (LGBM), Histogram-based Gradient Boosting (HGB), XGBoost (XGB), AdaBoost, Random Forest (RF), Multi-Layer Perceptron (MLP), Decision Tree (DT), logistic regression (LR), support vector machine (SVM), and K-nearest neighbor (KNN)^48^. This wide selection of algorithms was selected to evaluate their performance and suitability for the particular classification task at hand^49–51^.

LGBM, known for its speed and accuracy, was used to efficiently handle large data sets by creating unique vertical decision trees. Besides, calculating HGB uses histogram-based methods to optimize computing and memory usage, particularly useful for data structure^52,53^.

XGB, the versatile gradient worsting algorithm was also included because of its ability to handle complex data relationships^54^. AdaBoost is an ensemble method focusing on combining weak learners and it utilizes an iterative approach to improve classification accuracy^55^. RF is used for its robustness and flexibility for data types for classification and regression tasks^56^.

Furthermore, MLP, a neural network with multiple interconnected layers, was used to tackle complex, nonlinear data^57^. DT provided a straightforward yet powerful method for data partitioning based on key features, offering interpretability^58^. LR served as a simple yet effective baseline model, particularly suited for binary and multiclass classification tasks^59^.

SVM was leveraged for its versatility in handling high-dimensional data and linear or nonlinear classification tasks^60^. Finally, KNN offered an intuitive approach to classification, considering the majority class among the nearest neighbors of data points^61^.

The current study utilized Bayesian optimization with the Tree of Parzen Estimators (TPE) to optimize ML models, a powerful and efficient approach for hyperparameter tuning. Tree of Parzen Estimators is a probabilistic model-based optimization algorithm that effectively navigates the hyperparameter search space to find optimal configurations for ML models^62^.

TPE is particularly well-suited for hyperparameter optimization because it leverages a probabilistic model to make informed decisions about where to explore the hyperparameter space. Its working flow can be summaized as follows^62,63^:

- Modeling probability distributions: TPE begins by modeling the probability distributions of the hyperparameters. It maintains two distributions, one for promising configurations (i.e., exploitation) and another for less promising ones (i.e., exploration).
- Sampling: the algorithm then samples hyperparameters from these distributions. It does so in a way that favors promising regions based on the exploitation distribution but also explores other areas based on the exploration distribution.
- Evaluating the objective function: the sampled hyperparameters are used to train and evaluate the ML model using a chosen objective function, such as accuracy or loss. The performance of the model is recorded.
- Updating distributions: based on the performance of the sampled configuration, TPE updates the probability distributions for both exploitation and exploration. It allocates more samples to regions of the hyperparameter space that have shown promise.
- Iterative process: TPE iteratively repeats the process of sampling, evaluating, and updating distributions over a predefined number of iterations. This allows it to gradually refine its search and converge towards the optimal hyperparameters.
- Final configuration: at the end of the optimization process, TPE provides the best-found hyperparameters, which can then be used to train the final ML model.

TPE is known for its efficiency in finding near-optimal hyperparameter configurations with a relatively small number of model evaluations. It is particularly valuable when the hyperparameter space is high-dimensional or when manual tuning becomes impractical^63^. Table 1 presents the different hyperparameters for each ML classifier in the current study.

**Table 1:** The different hyperparameters for each utilized ML classifier in the current study.

Classifier	Hyperparameters
DT	Max depth (choice from range (1, # Features + 1)),
	Splitter (choice from [“Best”, “Random”]),
	Criterion (choice from [“Gini”, “Entropy”])
SVM	C (log normal (0, 1.0)),
	Kernel (choice from [“Linear”, “RBF”, “Poly”, “Sigmoid”]),
	Gamma (choice from [“Scale”, “Auto”]),
	Degree (choice from [1, 2, 3, 4, 5])
LR	C (log normal (0, 1.0)),
	Solver (choice from [“LibLinear”, “LBFGS”])
RF	Max depth (choice from range (1, # Features + 1)),
	# Estimators (choice from range (1, 100)),
	Criterion (choice from [“Gini”, “Entropy”])
KNN	# Neighbors (choice from range (1, int(# Samples / 2.0))),
	Weights (choice from [“Uniform”, “Distance”]),
	Algorithm (choice from [“Ball Tree”, “KDTree”, “Brute”]),
	Metric (choice from [“Minkowski”, “Euclidean”, “Manhattan”, “Chebyshev”])
LGBM	# Estimators (choice from range(1, 100)),
	Max depth (choice from range (1, # Features + 1)),
	Learning rate (log normal (0.01, 1.0))
XGB	Subsample (Uniform(0.1, 1)),
	# Estimators (choice from range (1, 100)),
	Max depth (choice from range (1, 51)),
	Learning rate (log normal (0.01, 1.0))
HGB	Max depth (choice from Range (1, # Features + 1)),
	Learning rate (log normal (0.01, 1.0))
AdaBoost	# Estimators (Choice from Range (1, 100)),
	Learning rate (log normal (0.01, 1.0))
CatBoost	# Estimators (choice from range (1, 100)),
	Early stopping rounds (choice from [2, 5, 8, 10, 50, 200])
MLP	Activation (choice from [“Logistic”, “ReLU”, “TanH”]),
	Learning rate (choice from [“Constant”, “Adaptive”, “Invscaling”]),
	Solver (choice from [“LBFGS”, “SGD”, “Adam”]),
	Hidden layer sizes (choice from range (16, 513, 16))

### Performance evaluation phase

In the present study, different performance metrics were used to evaluate the effectiveness of ML models in the AMD classification task. They play an important role in evaluating model quality and in determining decisions in model selection and optimization^50,64^.

Confusion matrix is a tabular representation of true positives (TP), true negatives (TN), false positives (FP), and false negatives (FN). It is important for evaluating model performance and deriving other metrics such as accuracy and F1^65–67^. Accuracy is a widely used metric that measures the average of correctly classified observations across all observations^68^. This provides a general understanding of the efficiency of the classification model, but can be misleading in cases of imbalanced datasets.

Sensitivity indicates how well the model is able to identify good patterns^69^. It calculates the proportion of true positives out of all actual positives. It is important when reducing false negatives is paramount, such as in clinical research^70^. Specificity examines how well the model is able to detect negative cases^71^. It calculates the proportion of true negatives out of all actual negatives. It is important when avoiding false positives is important, as seen in applications such as fraud detection^72^.

The receiver operating characteristic (ROC) is a graphical representation that illustrates a model’s performance across different thresholds^73^. It plots the TP rate against the FP rate at various threshold settings, with the area under the ROC curve (AUC-ROC) quantifying the model’s ability to distinguish between +ve and −ve instances^74,75^. BAC is an adjusted accuracy measure that is used for imbalanced datasets. It provides a more reliable performance assessment in skewed class distributions. Equations (4) to (10) presents the utilized metrics with their equations.

4 $$\begin{aligned}{} & {} \text {Accuracy} = \frac{\text {TP}+\text {TN}}{\text {TP}+\text {FP}+\text {FN}+\text {TN}} \end{aligned}$$

5 $$\begin{aligned}{} & {} \text {Specificity} = \frac{\text {TN}}{\text {FP}+\text {TN}} \end{aligned}$$

6 $$\begin{aligned}{} & {} \text {Recall (or Sensitivity)} = \frac{\text {TP}}{\text {TP}+\text {FN}} \end{aligned}$$

7 $$\begin{aligned}{} & {} \text {Precision} = \frac{\text {TP}}{\text {TP}+\text {FP}} \end{aligned}$$

8 $$\begin{aligned}{} & {} \text {ROC} = \frac{1}{\sqrt{2}} \times \sqrt{(\text {Sensitivity} ^ 2 + \text {Specificity} ^ 2)} \end{aligned}$$

9 $$\begin{aligned}{} & {} \text {F1-score} = \frac{2 \times (\text {Precision} \times \text {Recall})}{\text {Precision}+\text {Recall}} \end{aligned}$$

10 $$\begin{aligned}{} & {} \text {Balanced Accuracy (BAC)} = \frac{\text {Recall}+\text {Specificity}}{2} \end{aligned}$$

To reach a precise decision, a weighted sum metric (WSM) as presented in Eq. (11). It combines the mentioned performance metrics into a single comprehensive metric of model performance. This WSM is designed to consider the overall effectiveness of the models^76^.

11 $$\begin{aligned} \text {WSM} = w_1 \times \text {Accuracy} + w_2 \times \text {Sensitivity} + w_3 \times \text {Specificity} + w_4 \times \text {Precision} + w_5 \times \text {F1} + w_6 \times \text {ROC} + w_7 \times \text {BAC} \end{aligned}$$

By assigning weights to the individual metrics such as accuracy, sensitivity, and F1, the WSM can be aligned with the specific objectives of the classification task. In Eq. (11), $w_1$ to $w_7$ represent the weights assigned to each respective performance metric. This WSM provides a clear and interpretable way to balance trade-offs between different types of classification errors^77^. It enables decision-makers to make informed choices about the model performance that align with the study goals.

## Experiments

For all experiments, the reported performance metrics for the various ML models are: accuracy, sensitivity, specificity, precision, F1, ROC, BAC, and WSM. Also, as mentioned, The experimental protocols were approved by the authors’ and patients’ institutions: University of Louisville and Mansoura University.

Table 2 presents the performance results of the implemented framework across different phases, each corresponding to a mask positioned at varying distances from 0 to 1500. The distances are measured in units that align with the dimensions of the mask. The table provides a detailed overview of various evaluation metrics for each configuration, shedding light on the framework’s efficacy under different spatial settings. The “Distance” column specifies the distance of the mask from its original position, and the “Combinations” column denotes the specific combinations of parameters used in each experiment.

The subsequent columns contain performance metrics, including Accuracy (ACC), Sensitivity (SNS), Specificity (SPC), Precision (PRC), F1 score (F1), Receiver Operating Characteristic (ROC) score, Balanced Accuracy (BAC), and Weighted Similarity Metric (WSM). Examining the results, notable trends emerge. For instance, as the distance increases, there is a discernible impact on metrics such as accuracy, sensitivity, and specificity.

The table indicates that the performance varies across different combinations of parameters and distances. Notably, at 1500 units, the framework achieves impressive results across all metrics, indicating its robust performance when the mask is positioned farther from its original location. Tables 5, 6, 7, 8, 9, 10 and 11 in the Appendices present the inner details of each row/distance.

**Table 2:** The performance results obtained by implementing the framework phases with the mask positioned at a polygon distanced from 0 to 1500, which corresponds to the mask itself, are presented.

Distance	Combinations	ACC	SNS	SPC	PRC	F1	ROC	BAC	WSM
0	Top-7	90.45	80.92	93.62	82.84	81.26	87.63	87.27	86.29
250	Top-10	90.50	81.04	93.65	81.71	81.03	87.70	87.35	86.14
500	Top-7	90.14	80.33	93.42	80.67	80.27	87.24	86.87	85.56
750	Top-8	89.30	78.67	92.86	78.95	78.65	86.17	85.77	84.34
1000	Top-9	90.20	80.45	93.46	82.36	79.68	87.88	86.96	85.86
1250	Top-2	93.22	86.49	95.48	87.09	86.20	91.35	90.98	90.12
1500	Top-3	96.31	92.65	97.54	92.75	92.54	95.21	95.10	94.59

The experiment conducted at a distance of 1500 units stands out as the most noteworthy configuration in Table 2. In this setting, the mask is positioned at a considerable distance from its original location, and the framework achieves outstanding performance across all evaluated metrics. With an impressive accuracy (ACC) of 96.31%, the model demonstrates a high degree of correctness in its predictions. Moreover, the sensitivity (SNS) and specificity (SPC) scores, measuring the model’s ability to identify positive and negative instances, are notably high at 92.65% and 97.54%, respectively. The Precision (PRC) and F1 score (F1) further highlight the framework’s precision and balance between precision and recall. The receiver operating characteristic (ROC) score, balanced accuracy (BAC), and weighted similarity Metric (WSM) collectively reinforce the exceptional performance of the model in accurately classifying instances when the mask is positioned at this substantial distance. This result underscores the model’s resilience and effectiveness, particularly when faced with spatial variations in the positioning of the mask.

Majority voting: by applying the weighted majority voting on the best classifiers regarding each polygon distanced from 0 to 1500, the performance is enhanced with an overall accuracy of 96.85%, sensitivity of 93.72%, specificity of 97.89%, precision of 93.86%, F1 score of 93.72%, ROC of 95.85%, BAC of 95.81%, and WSM of 95.38%. The fact that the performance is enhanced by applying majority voting on the best classifiers regarding each polygon distanced from 0 to 1500 suggests that the model is able to learn different patterns for different polygon distances. This is important as the model is more likely to be able to generalize to new instances, even if the polygon distances in the new instances are different from the polygon distances in the training instances.

Enhancing interpretation through contour overlay: in image analysis and classification, the overlay of contours on an image plays an important role in improving the interpretation of the results. This method is particularly valuable when dealing with multiple classes, each of which is assigned a specific label.

**Figure 5:**
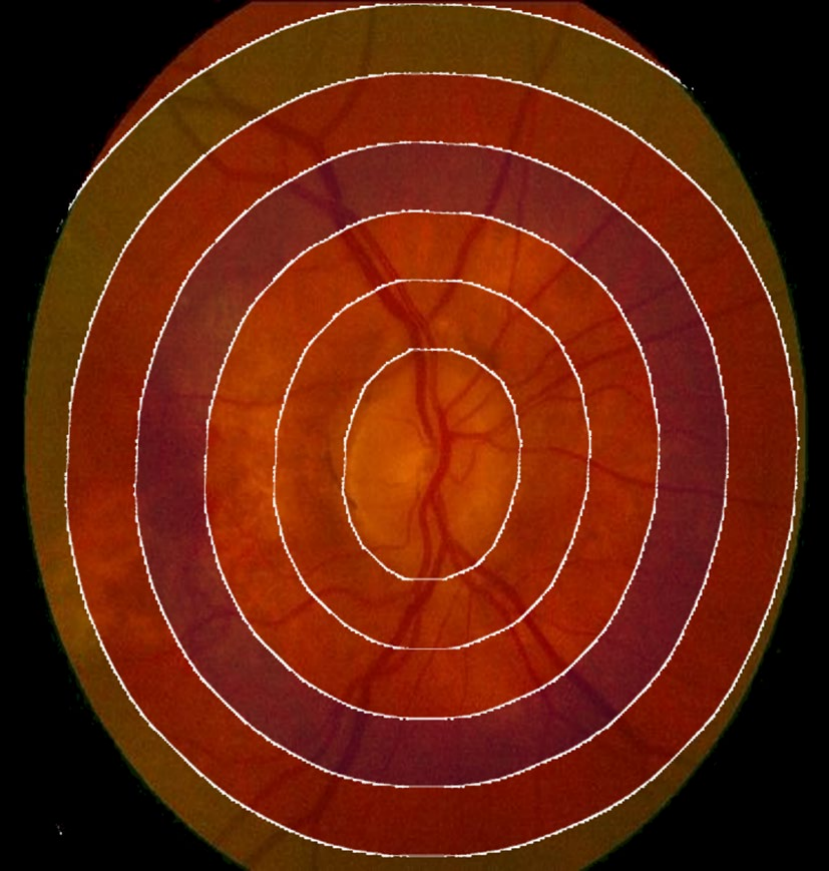
Visualization of an image with overlaid contours, where most of them are correctly identified, except for two. The Wet class is depicted in red, the GA class in green, and the Normal class in yellow, all with an opacity set to 0.1. White contours have been incorporated to enhance the visualization.

Figure 5 displays the overlaid contours, where most of them are correctly identified, except for two. The Wet class is depicted in red, the GA class in green, and the Normal class in yellow, all with an opacity set to 0.1. This visual representation allows for a quick and intuitive assessment of which contours have been inaccurately diagnosed. By assigning distinct colors to each class, it becomes evident which specific classes are affected by misclassifications.

**Figure 6:**
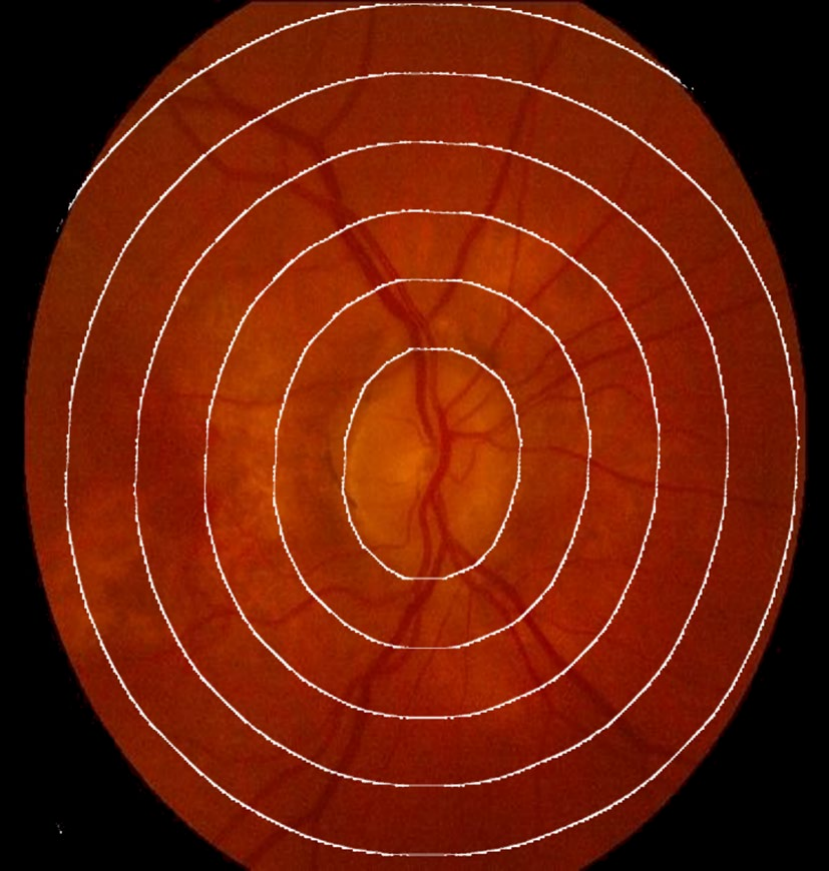
Visualization of an overlaid image featuring the Wet class with accurately diagnosed contours. The overlay is in red with an opacity of 0.1. White contours have been incorporated to enhance the visualization.

While the visual identification of incorrectly diagnosed contours is vital, it is also essential to consider the broader context. Figure 6 demonstrates an overlaid image with different contours, all correctly diagnosed. This comprehensive visualization not only assures the accuracy of individual contours but also provides confidence in the overall diagnosis of the entire image.

## Overall discussion

In our study, we have presented a detailed Computer-Aided Diagnosis (CAD) framework for Age-related Macular Degeneration (AMD) analysis. The CAD system is designed to assist in the diagnosis of AMD through a multi-stage process involving data acquisition, pre-processing, feature extraction, scaling, classification, and optimization. The entire methodology is encapsulated within a systematic framework, as illustrated in Fig. 1.

The framework begins with the data acquisition phase, where necessary information is obtained. This is followed by the pre-processing stage, where various techniques such as Average CDF calculation, CLAHE enhancements, interpolation, ROI extraction, and contour analysis are applied to improve data quality and prepare it for analysis. The feature extraction phase involves extracting meaningful patterns from the pre-processed data using Gray-Level Co-occurrence Matrix (GLCM) and Gray-Level Run-Length Matrix (GLRLM) methods. This step is crucial in quantifying the characteristics of the images.

In our research, the choice of feature extraction methods was driven by a consideration of the unique characteristics of the data and the specific requirements of the AMD diagnosis task. For capturing global appearance markers from the entire fundus image, we opted for techniques such as average cumulative distribution function (ACDF) calculation and contrast limited adaptive histogram equalization (CLAHE). These methods are adept at providing an understanding of pixel intensity distributions and enhancing contrast throughout the entire fundus image. This global perspective is essential for ensuring that the diagnostic model considers the overall structure and characteristics of the retina.

For local appearance markers, particularly in the optical disc section, we employed techniques such as region of interest (ROI) extraction and contour analysis. By focusing on specific regions of interest within the image and using binary masks and contour analysis, we aimed to capture detailed local information. This emphasis on local features is critical for identifying subtle patterns or abnormalities around the optical disc, contributing to a nuanced and accurate AMD diagnosis.

After feature extraction, the data goes through a scaling phase, where different scaling techniques such as standardization, min-max scaling, and max absolute scaling are applied to ensure that features are on compatible scales, optimizing the behavior of various machine learning (ML) algorithms.

The heart of the CAD system lies in the classification and optimization phase, where a variety of ML algorithms, including LightGBM, Histogram-based Gradient Boosting, XGBoost, AdaBoost, Random Forest, Multi-Layer Perceptron, Decision Tree, Logistic Regression, Support Vector Machine, and K-nearest neighbor, are employed. The Tree of Parzen Estimators (TPE) is used for hyperparameter optimization, ensuring the ML models are finely tuned for the specific task.

Finally, the performance evaluation phase employs various metrics such as confusion matrix, accuracy, sensitivity, specificity, ROC, F1-score, and balanced accuracy (BAC) to comprehensively evaluate the effectiveness of the ML models in AMD classification. These metrics are not only presented individually but are also combined into a Weighted Sum Metric (WSM) to provide an insightful measure of model performance.

Concerning the consideration of using transformers and convolutions for feature extraction, it is important to note that our decision was influenced by several factors. In the context of medical imaging, obtaining large labeled datasets for training deep learning models can be challenging. Conventional methods remain robust and effective even with smaller datasets. Additionally, the interpretability of results is a crucial consideration in medical contexts. The use of ACDF, CLAHE, and contour analysis provides transparency and interpretability, allowing healthcare professionals to understand the features contributing to a diagnosis. Furthermore, computational efficiency is a key factor, and deep learning models, particularly those involving Transformers and convolutions, may demand substantial computational resources. The simplicity of our chosen methods ensures computational efficiency without compromising the diagnostic accuracy required for AMD diagnosis. While deep learning approaches have shown success in various domains, the specific requirements of our AMD diagnosis task, including interpretability, dataset size considerations, and computational efficiency, led us to choose the outlined feature extraction methods.

## Limitations

Several limitations should be noted in this study. The effectiveness of the CAD system relies on data quality and quantity, including variations in image quality and demographic representation, potentially affecting its generalizability. Labeling fundus images with AMD stages can introduce inter-observer variability, impacting ML model training and evaluation. Additionally, the study primarily focuses on broad AMD stage classification, excluding finer subtypes. External validation in diverse clinical settings is necessary to confirm real-world applicability. Regulatory approvals, clinical integration, and addressing ethical concerns are essential for the CAD system’s responsible deployment. Deep learning models lack transparency, and the CAD system should always complement clinical expertise. Long-term follow-up and adaptation to geographic variability are areas for future exploration.

Despite these limitations, this research represents a significant step towards enhancing AMD diagnosis through ML. Addressing these challenges and conducting further research can contribute to the continued improvement and responsible implementation of AI-driven diagnostic tools for AMD.

## Conclusions and future directions

This study has critically examined the effective application of ML methods to accurately classify the AMD stage from fundus images All aspects of AMD diagnosis are discussed, from data acquisition to preprocessing to feature extraction and selection of ML algorithms therefore. The goal was to develop a non-invasive CAD system that maximizes the accuracy and clinical utility of AMD classification. By applying weighted majority voting on the best classifiers, the performance is enhanced with an overall accuracy of 96.85%, sensitivity of 93.72%, specificity of 97.89%, precision of 93.86%, F1 score of 93.72%, ROC of 95.85%, BAC of 95.81%, and WSM of 95.38%. These results suggest a successful CAD system that can play an important role in the early detection and diagnosis of AMD.

One of the noteworthy outcomes of this study is the improvement in the classification of AMD stages. Through rigorous experimentation and analysis, an advance in the accuracy and reliability of categorizing AMD into geographic atrophy (GA), intermediate AMD, normal, and wet AMD categories has been achieved. This enhanced precision is a critical step towards facilitating appropriate treatment strategies for patients. Furthermore, intricate patterns and relationships within fundus images have been illuminated by the study. These patterns enable the identification of subtle indicators of different AMD phases, which in turn can aid in early intervention and treatment. Through the automation of the AMD diagnosis process and the reduction of inter-observer discrepancies, enhanced patient care is facilitated by our CAD system.

Looking ahead, several promising directions for future research in this field can be explored. Firstly, further optimization of the ML models can be investigated. Techniques like hyperparameter tuning and the integration of more advanced techniques such as transformers and deep learning can potentially boost the system’s accuracy even further. Additionally, the scalability and applicability of the CAD system to larger datasets and diverse populations can be explored. Robustness across different demographic groups and geographic regions can ensure its broad clinical utility. Moreover, the performance of the CAD system can be enhanced by the incorporation of more extensive image datasets and advanced imaging technologies. This could involve the utilization of high-resolution images and multimodal data fusion for a more comprehensive assessment. In terms of clinical application, validation studies involving real-world patient data and collaboration with healthcare institutions can validate the effectiveness of the CAD system in a clinical setting. Regulatory approval and integration into routine clinical practice would be significant milestones. Lastly, ongoing research into the underlying mechanisms of AMD, including genetics, biomarkers, and treatment options, can complement the diagnostic capabilities of the CAD system. Combining ML-based diagnosis with cutting-edge treatment strategies can usher in a new era of precision medicine for AMD.

## Data Availability

Access to the dataset used in this study is subject to availability and can be made available upon request for study and research purposes. Researchers interested in gaining access to the dataset for further research are encouraged to contact the authors. The datasets generated during and/or analyzed during the current study are available from the corresponding author on a reasonable request. The data, figures, and scripts are licensed under CC-BY-NC-ND (or Creative Commons Attribution NonCommercial NoDerivs) from the time we start working on them until the document is published.

## Appendix

### First and second-order features

The first- and second-order features were presented in Tables 3 and 4, respectively. The presented first-order features capture essential statistical properties of pixel intensities within ROIs and distance maps. These features, including mean, standard deviation, median, and others, provide a comprehensive description of the distribution and variation in pixel values. Moving to second-order features, these metrics, based on gray-level run-length matrices (GLRLM), delve deeper into the spatial relationships between pixel intensities. The energy, entropy, contrast, and other measures derived from GLRLM offer insights into the texture patterns of the images.

**Table 3:** The equations employed for calculating the first-order-based markers.

First-order feature	Equation
Mean	$\mu = \frac{1}{N} \sum _{i=1}^{N} x_i$
Standard deviation	$\sigma = \sqrt{\frac{1}{N} \sum _{i=1}^{N} (x_i - \mu )^2}$
Median	${\tilde{x}} = \text {median}(x)$ (for odd N)
Median	${\tilde{x}}_L = \text {median}(x[:\frac{N}{2}])$, ${\tilde{x}}_U = \text {median}(x[\frac{N}{2}:])$ (for even N)
Mean absolute deviation	$\text {MAD} = \frac{1}{N} \sum _{i=1}^{N} |x_i - \mu |$
Robust mean absolute deviation	$\text {RMAD} = \text {median}(|x_i - \text {median}(x)|)$
Median absolute deviation	$\text {MAD} = \text {median}(|x_i - \text {median}(x)|)$
Root mean square	$\text {RMS} = \sqrt{\frac{1}{N} \sum _{i=1}^{N} x_i^2}$
Variance	$\sigma ^2 = \frac{1}{N} \sum _{i=1}^{N} (x_i - \mu )^2$
Minimum	$\text {Min} = \min (x_1, x_2, \ldots , x_N)$
Maximum	$\text {Max} = \max (x_1, x_2, \ldots , x_N)$
Skewness	$\text {Skew} = \frac{\frac{1}{N} \sum _{i=1}^{N} (x_i - \mu )^3}{\sigma ^3}$
Kurtosis	$\text {Kurt} = \frac{\frac{1}{N} \sum _{i=1}^{N} (x_i - \mu )^4}{\sigma ^4}$
Shannon entropy	$H(X) = -\sum _{i=1}^{N} p(x_i) \log _2(p(x_i))$
Interquartile range	$\text {IQR} = Q_3 - Q_1$

They are used to quantify different aspects of texture patterns in the ROIs and distance maps.

**Table 4:** The equations employed for calculating the second-order-based GLRLM markers.

Second-order feature	Equation
Energy	$\text {Energy} = \sum _{i=1}^{N} \sum _{j=1}^{N} \times \text {GLCM}(i, j)^2$
Entropy	$-\sum _{i=1}^{N} \sum _{j=1}^{N} \times \text {GLCM}(i, j) \times \log (\text {GLCM}(i, j) + \epsilon )$
Contrast	$\sum _{i=1}^{N} \sum _{j=1}^{N} (i - j)^2 \times \text {GLCM}(i, j)$
Correlation	$\frac{\sum _{i=1}^{N} \sum _{j=1}^{N} (i - \mu _x)(j - \mu _y) \times \text {GLCM}(i, j)}{\sigma _x \sigma _y}$
Homogeneity	$\sum _{i=1}^{N} \sum _{j=1}^{N} \times \frac{\text {GLCM}(i, j)}{1 + |i - j|}$
Angular second moment (ASM)	$\sum _{i=1}^{N} \sum _{j=1}^{N} \text {GLCM}(i, j)^2$
Dissimilarity	$\sum _{i=1}^{N} \sum _{j=1}^{N} |i - j| \times \text {GLCM}(i, j)$
Inverse difference (ID)	$\sum _{i=1}^{N} \sum _{j=1}^{N} \frac{\text {GLCM}(i, j)}{1 + (i - j)^2}$
Cluster shade	$\sum _{i=1}^{N} \sum _{j=1}^{N} (i + j - \mu _x - \mu _y)^3 \times \text {GLCM}(i, j)$
Cluster prominence	$\sum _{i=1}^{N} \sum _{j=1}^{N} (i + j - \mu _x - \mu _y)^4 \times \text {GLCM}(i, j)$
Max. probability	$\max (\text {GLCM}(i, j))$
SRE (short run emphasis)	$\sum _{\text {length}=1}^{N} \sum _{\text {intensity}=1}^{N} \frac{\Psi (\text {length}, \text {intensity})}{\text {length}^2}$
LRE (long run emphasis)	$\sum _{\text {length}=1}^{N} \sum _{\text {intensity}=1}^{N} \Psi (\text {length}, \text {intensity}) \cdot \text {length}^2$
GLN (gray-level nonuniformity)	$\sum _{\text {length}=1}^{N} \sum _{\text {intensity}=1}^{N} \Psi (\text {length}, \text {intensity})^2$
RLN (run-length nonuniformity)	$\frac{\sum _{\text {length}=1}^{N} \sum _{\text {intensity}=1}^{N} \Psi (\text {length}, \text {intensity})^2}{\sum _{\text {length}=1}^{N} \sum _{\text {intensity}=1}^{N} \Psi (\text {length}, \text {intensity})}$
RP (run percentage)	$\sum _{\text {intensity}=1}^{N} \left( \frac{\sum _{\text {length}=1}^{N} \Psi (\text {length}, \text {intensity})}{\text {length}}\right)$
LGLRE (low gray-level run emphasis)	$\sum _{\text {length}=1}^{N} \sum _{\text {intensity}=1}^{N} \frac{\Psi (\text {length}, \text {intensity})}{\text {intensity}^2}$
HGLRE (high gray-level run emphasis)	$\sum _{\text {length}=1}^{N} \sum _{\text {intensity}=1}^{N} \Psi (\text {length}, \text {intensity}) \cdot \text {intensity}^2$
SRLGLE (short run low gray-level emphasis)	$\sum _{\text {length}=1}^{N} \sum _{\text {intensity}=1}^{N} \frac{\Psi (\text {length}, \text {intensity})}{\text {length} \cdot \text {intensity}^2}$
SRHGLE (short run high gray-level emphasis)	$\sum _{\text {length}=1}^{N} \sum _{\text {intensity}=1}^{N} \frac{\Psi (\text {length}, \text {intensity})}{\text {length}^2 \cdot \text {intensity}}$
LRLGLE (long run low gray-level emphasis)	$\sum _{\text {length}=1}^{N} \sum _{\text {intensity}=1}^{N} \frac{\Psi (\text {length}, \text {intensity}) \cdot \text {length}^2}{\text {intensity}^2}$
LRHGLE (long run high gray-level emphasis)	$\sum _{\text {length}=1}^{N} \sum _{\text {intensity}=1}^{N} \frac{\Psi (\text {length}, \text {intensity}) \cdot \text {length}^2}{\text {intensity}}$

They are used to quantify different aspects of texture patterns in the ROIs and distance maps.

### Detailed experiments

Table 5 shows the performance results obtained using the proposed framework with with a mask at polygon distance 0. The results show that both LGBM and Top-7 perform very well, with accuracies above 90%. However, Top-7 slightly outperforms LGBM. Top-7 has a higher accuracy (90.33% vs 90.45%), sensitivity (80.69% vs 80.92%), specificity (93.53% vs 93.62%), precision (82.78% vs 82.84%), F1-score (81.08% vs 81.26%), WSM (86.14% vs 86.29%), and BAC (87.11% vs 87.27%). Both LGBM and Top-7 achieve ROC-AUC scores above 0.8, indicating that they are both good classifiers for this task. Both LGBM and Top-7 achieve high BAC scores, indicating that they are both robust classifiers.

**Table 5:** The performance results obtained by implementing the framework phases with the mask positioned at a polygon distanced of 0, which corresponds to the mask itself, are presented.

ML	Accuracy	Sensitivity	Specificity	Precision	F1	ROC	BAC	WSM
LGBM	90.33	80.69	93.53	82.78	81.08	87.47	87.11	86.14
HGB	90.27	80.57	93.49	82.73	80.93	87.42	87.03	86.06
XGB	89.56	79.15	93.02	81.13	79.55	86.46	86.08	84.99
AdaBoost	79.83	59.72	86.50	64.55	60.19	74.91	73.11	71.26
RF	89.09	78.20	92.72	79.62	78.40	85.88	85.46	84.20
MLP	89.62	79.27	93.08	79.84	79.43	86.46	86.17	84.84
DT	87.19	74.41	91.45	76.27	74.65	83.54	82.93	81.49
LR	82.86	65.76	88.58	65.90	65.70	78.12	77.17	74.87
SVM	81.45	62.91	87.63	63.93	63.08	76.36	75.27	72.95
KNN	80.92	61.85	87.29	62.81	62.01	75.70	74.57	72.16
Top-2	90.33	80.69	93.53	82.78	81.08	87.47	87.11	86.14
Top-3	90.03	80.09	93.33	82.19	80.49	87.09	86.71	85.71
Top-4	90.03	80.09	93.33	82.19	80.49	87.09	86.71	85.71
Top-5	90.09	80.21	93.38	82.33	80.61	87.17	86.80	85.80
Top-6	90.03	80.09	93.34	82.33	80.51	87.10	86.72	85.73
Top-7	90.45	80.92	93.62	82.84	81.26	87.63	87.27	86.29
Top-8	90.27	80.57	93.50	82.56	80.92	87.41	87.04	86.04
Top-9	90.27	80.57	93.50	82.50	80.92	87.39	87.04	86.03
Top-10	90.04	80.09	93.34	82.15	80.42	87.12	86.72	85.70

Table 6 shows the performance results obtained using the proposed framework with with a mask at polygon distance 250. Both LGBM and Top-10 perform very well, with accuracies above 90%. However, Top-10 outperforms LGBM. Top-10 has a higher accuracy (90.50% vs 90.20%), sensitivity (81.04% vs 80.45%), specificity (93.65% vs 93.46%), precision (81.71% vs 81.48%), F1-score (81.03% vs 80.48%), ROC (87.70% vs 87.35%), BAC (86.95% vs 87.35%), and WSM (86.14% vs 85.77%). If you need the highest possible accuracy and robustness, then Top-10 is the better choice. The difference in ROC between Top-10 and LGBM is also very small (0.35%). The difference in BAC between Top-10 and LGBM is slightly larger (0.40%).

**Table 6:** The performance results obtained by implementing the framework phases with the mask positioned at a polygon distanced of 250.

ML	Accuracy	Sensitivity	Specificity	Precision	F1	ROC	BAC	WSM
LGBM	90.20	80.45	93.46	81.48	80.48	87.35	86.95	85.77
HGB	89.79	79.62	93.18	80.90	79.69	86.84	86.40	85.20
XGB	89.61	79.27	93.06	80.40	79.36	86.60	86.16	84.92
AdaBoost	77.80	55.69	85.16	59.26	56.51	72.03	70.43	68.13
RF	89.50	79.03	92.99	79.25	78.90	86.42	86.01	84.58
MLP	88.48	77.01	92.30	77.68	77.13	85.07	84.66	83.19
DT	87.00	74.05	91.32	75.42	74.23	83.30	82.69	81.14
LR	82.07	64.22	88.03	65.29	64.46	77.16	76.13	73.91
SVM	79.88	59.83	86.56	61.41	59.39	75.10	73.20	70.77
KNN	81.49	63.03	87.64	63.48	62.88	76.58	75.34	72.92
Top-2	90.20	80.45	93.46	81.48	80.48	87.35	86.95	85.77
Top-3	90.08	80.21	93.38	81.38	80.28	87.20	86.79	85.62
Top-4	90.20	80.45	93.46	81.36	80.51	87.32	86.95	85.75
Top-5	90.03	80.09	93.34	81.03	80.15	87.10	86.72	85.49
Top-6	90.02	80.09	93.34	81.09	80.17	87.10	86.72	85.50
Top-7	90.38	80.81	93.57	81.80	80.88	87.56	87.19	86.03
Top-8	90.09	80.21	93.38	81.13	80.25	87.18	86.79	85.58
Top-9	90.38	80.81	93.57	81.46	80.79	87.55	87.19	85.97
Top-10	90.50	81.04	93.65	81.71	81.03	87.70	87.35	86.14

Table 7 shows the performance results obtained using the proposed framework with with a mask at polygon distance 500. Both XGB and Top-7 perform very well, with accuracies above 90%. However, Top-7 outperforms XGB. Top-7 has a higher accuracy (90.14% vs 89.78%), sensitivity (80.33% vs 79.62%), specificity (93.42% vs 93.18%), precision (80.67% vs 79.85%), BAC (86.40% vs 86.87%), F1-score (80.27% vs 79.62%), ROC (87.24% vs 86.74%), and WSM (85.56% vs 85.03%). If you need the highest possible accuracy and robustness, then Top-7 is the better choice. The difference in ROC between Top-7 and XGB is also very small (0.50%). The difference in BAC between Top-7 and XGB is slightly larger (0.47%).

**Table 7:** The performance results obtained by implementing the framework phases with the mask positioned at a polygon distanced of 500.

ML	Accuracy	Sensitivity	Specificity	Precision	F1	ROC	BAC	WSM
LGBM	89.25	78.55	92.82	78.74	78.48	86.11	85.69	84.23
HGB	89.25	78.55	92.83	78.61	78.47	86.09	85.69	84.21
XGB	89.78	79.62	93.18	79.85	79.62	86.74	86.40	85.03
AdaBoost	80.36	60.78	86.88	63.21	61.43	75.02	73.83	71.64
RF	88.95	77.96	92.63	79.20	77.64	86.01	85.30	83.96
MLP	88.24	76.54	92.15	76.76	76.51	84.80	84.34	82.76
DT	88.01	76.07	92.00	76.23	75.75	84.66	84.03	82.39
LR	81.59	63.27	87.72	63.30	63.23	76.64	75.50	73.03
SVM	81.18	62.44	87.45	62.50	60.99	77.03	74.95	72.36
KNN	81.20	62.44	87.49	61.82	61.78	76.43	74.96	72.30
Top-2	89.78	79.62	93.18	79.85	79.62	86.74	86.40	85.03
Top-3	89.48	79.03	92.98	79.21	78.99	86.39	86.00	84.58
Top-4	89.84	79.74	93.22	79.93	79.69	86.84	86.48	85.11
Top-5	89.78	79.62	93.18	79.83	79.57	86.77	86.40	85.02
Top-6	90.02	80.09	93.34	80.32	80.01	87.10	86.72	85.37
Top-7	90.14	80.33	93.42	80.67	80.27	87.24	86.87	85.56
Top-8	89.84	79.74	93.22	80.01	79.63	86.88	86.48	85.11
Top-9	89.60	79.27	93.06	79.46	79.16	86.57	86.16	84.76
Top-10	89.78	79.62	93.18	79.81	79.55	86.78	86.40	85.02

Table 8 shows the performance results obtained using the proposed framework with with a mask at polygon distance 750. Both HGB and Top-8 perform well, with accuracies above 88%. However, Top-8 outperforms HGB. Top-8 has a higher accuracy (89.30% vs 88.53%), sensitivity (78.67% vs 77.13%), specificity (92.86% vs 92.35%), precision (78.95% vs 77.28%), F1-score (78.65% vs 77.19%), ROC (86.17% vs 85.13%), BAC (85.77% vs 84.74%), and WSM (83.19% vs 84.34%). The difference in accuracy between Top-8 and HGB is only 0.77%. This is a relatively small difference. The difference in ROC between Top-8 and HGB is also relatively small (1.04%). The difference in BAC between Top-8 and HGB is slightly larger (1.03%). The difference in WSM between Top-8 and HGB is also relatively small (1.15%).

**Table 8:** The performance results obtained by implementing the framework phases with the mask positioned at a polygon distanced of 750.

ML	Accuracy	Sensitivity	Specificity	Precision	F1	ROC	BAC	WSM
LGBM	87.64	75.36	91.75	75.59	75.40	84.05	83.55	81.90
HGB	88.53	77.13	92.35	77.28	77.19	85.13	84.74	83.19
XGB	88.36	76.78	92.23	76.85	76.78	84.92	84.50	82.92
AdaBoost	79.29	58.65	86.17	61.88	59.06	74.01	72.41	70.21
RF	88.42	76.90	92.28	77.46	76.71	85.16	84.59	83.07
MLP	87.46	75.00	91.63	75.78	74.80	84.05	83.31	81.72
DT	85.51	71.09	90.34	71.00	70.52	81.72	80.71	78.70
LR	83.85	67.77	89.24	67.44	67.17	79.65	78.51	76.24
SVM	79.75	59.60	86.50	60.69	57.92	75.60	73.05	70.44
KNN	80.94	61.97	87.29	61.91	61.59	76.07	74.63	72.06
Top-2	88.53	77.13	92.35	77.28	77.19	85.13	84.74	83.19
Top-3	88.95	77.96	92.63	77.94	77.91	85.69	85.29	83.77
Top-4	88.83	77.73	92.55	77.71	77.66	85.55	85.14	83.59
Top-5	89.01	78.08	92.66	78.16	78.05	85.77	85.37	83.87
Top-6	89.07	78.20	92.70	78.29	78.17	85.85	85.45	83.96
Top-7	89.07	78.20	92.70	78.40	78.16	85.87	85.45	83.98
Top-8	89.30	78.67	92.86	78.95	78.65	86.17	85.77	84.34
Top-9	89.13	78.32	92.74	78.54	78.20	85.99	85.53	84.06
Top-10	89.07	78.20	92.70	78.36	78.14	85.87	85.45	83.97

Table 9 shows the performance results obtained using the proposed framework with with a mask at polygon distance 1000. Both LGBM and Top-9 perform very well, with accuracies above 90%. However, LGBM slightly outperforms Top-9 on all metrics except for precision, ROC, and WSM, where Top-9 is slightly better. LGBM has a higher accuracy (90.25% vs 90.20%), sensitivity (80.57% vs 80.45%), specificity (93.50% vs 93.46%), F1-score (79.97% vs 79.68%), and BAC (87.03% vs 86.96%). Top-9 achieves a slightly higher WSM score than LGBM (85.86% vs 85.83%). Overall, LGBM is the better performing algorithm, but the difference in performance between LGBM and Top-9 is very small. If you need the highest possible accuracy and robustness, then LGBM is the better choice. However, if WSM is more important to you, then Top-9 may be the better choice. The difference in accuracy between LGBM and Top-9 is only 0.05%. This is a very small difference. The difference in ROC between LGBM and Top-9 is also very small (0.07%). The difference in BAC between LGBM and Top-9 is slightly larger (0.07%). The difference in WSM between LGBM and Top-9 is also very small (0.03%).

**Table 9:** The performance results obtained by implementing the framework phases with the mask positioned at a polygon distanced of 1000.

ML	Accuracy	Sensitivity	Specificity	Precision	F1	ROC	BAC	WSM
LGBM	90.25	80.57	93.50	81.67	79.97	87.81	87.03	85.83
HGB	90.13	80.33	93.42	80.89	79.93	87.48	86.87	85.58
XGB	88.89	77.84	92.60	78.09	77.35	85.90	85.22	83.70
AdaBoost	83.43	66.94	88.95	67.62	66.50	79.25	77.95	75.81
RF	88.25	76.54	92.19	77.94	75.66	85.35	84.36	82.90
MLP	87.81	75.71	91.87	78.54	74.48	85.27	83.79	82.50
DT	88.43	76.90	92.30	78.50	76.24	85.54	84.60	83.22
LR	83.73	67.54	89.16	68.09	65.51	80.41	78.35	76.11
SVM	82.90	65.88	88.59	68.65	62.92	80.05	77.24	75.17
KNN	83.09	66.23	88.75	65.52	65.39	78.85	77.49	75.05
Top-2	90.25	80.57	93.50	81.67	79.97	87.81	87.03	85.83
Top-3	90.19	80.45	93.46	81.10	79.94	87.63	86.95	85.68
Top-4	90.19	80.45	93.46	81.06	79.93	87.63	86.95	85.67
Top-5	89.72	79.50	93.15	80.07	78.94	87.02	86.33	84.96
Top-6	89.84	79.74	93.22	80.65	79.12	87.26	86.48	85.19
Top-7	89.84	79.74	93.22	80.87	79.02	87.33	86.48	85.21
Top-8	89.96	79.98	93.31	81.07	79.30	87.45	86.64	85.39
Top-9	90.20	80.45	93.46	82.36	79.68	87.88	86.96	85.86
Top-10	90.02	80.09	93.34	81.56	79.35	87.62	86.72	85.53

Table 10 shows the performance results obtained using the proposed framework with with a mask at polygon distance 1250. Both LGBM and Top-2 perform extremely well, with accuracies above 93%. They achieve identical results on all metrics, including sensitivity, specificity, precision, F1-score, ROC, BAC, and WSM.

**Table 10:** The performance results obtained by implementing the framework phases with the mask positioned at a polygon distanced of 1250.

ML	Accuracy	Sensitivity	Specificity	Precision	F1	ROC	BAC	WSM
LGBM	93.22	86.49	95.48	87.09	86.20	91.35	90.98	90.12
HGB	92.93	85.90	95.28	86.70	85.59	91.01	90.59	89.71
XGB	92.52	85.07	95.01	85.61	84.73	90.44	90.04	89.06
AdaBoost	82.91	65.88	88.62	67.85	64.92	79.03	77.25	75.21
RF	91.27	82.58	94.17	84.58	82.05	89.08	88.38	87.44
MLP	91.14	82.35	94.09	83.01	81.67	88.91	88.22	87.06
DT	91.21	82.46	94.13	83.79	81.87	88.98	88.30	87.25
LR	87.05	74.17	91.37	74.74	72.57	84.25	82.77	80.99
SVM	87.29	74.64	91.52	77.58	73.53	84.64	83.08	81.75
KNN	87.47	75.00	91.65	75.04	74.37	84.18	83.32	81.58
Top-2	93.22	86.49	95.48	87.09	86.20	91.35	90.98	90.12
Top-3	92.93	85.90	95.28	86.52	85.63	90.96	90.59	89.69
Top-4	92.87	85.78	95.24	86.50	85.50	90.90	90.51	89.61
Top-5	92.75	85.55	95.16	86.52	85.24	90.79	90.35	89.48
Top-6	92.81	85.66	95.20	86.54	85.36	90.85	90.43	89.55
Top-7	92.81	85.66	95.20	86.67	85.32	90.89	90.43	89.57
Top-8	92.93	85.90	95.28	87.00	85.57	91.04	90.59	89.76
Top-9	92.87	85.78	95.24	87.01	85.41	90.99	90.51	89.69
Top-10	92.57	85.19	95.04	86.31	84.80	90.62	90.12	89.24

Table 11 shows the performance results obtained using the proposed framework with with a mask at polygon distance 1500. Both MLP and Top-3 perform extremely well, with accuracies above 96%. Top-3 outperforms MLP. Top-3 has a higher accuracy (96.31% vs 96.08%), sensitivity (92.65% vs 92.18%), specificity (97.54% vs 97.39%), precision (92.75% vs 92.32%), F1-score (92.54% vs 91.98%), ROC (95.21% vs 94.94%), BAC (94.78% vs 95.10%), and WSM (94.59% vs 94.24%). If you need the highest possible accuracy and robustness, then Top-3 is the better choice. The difference in accuracy between Top-3 and MLP is only 0.23%. This is a very small difference, and it is possible that it is due to chance. The difference in ROC between Top-3 and MLP is also very small (0.27%). The difference in BAC between Top-3 and MLP is slightly larger (0.32%). The difference in WSM between Top-3 and MLP is also very small (0.35%).

**Table 11:** The performance results obtained by implementing the framework phases with the mask positioned at a polygon distanced of 1500.

ML	Accuracy	Sensitivity	Specificity	Precision	F1	ROC	BAC	WSM
LGBM	95.72	91.47	97.14	91.66	91.35	94.45	94.31	93.73
HGB	96.08	92.18	97.38	92.27	92.04	94.91	94.78	94.23
XGB	95.31	90.64	96.87	90.99	90.40	93.98	93.75	93.13
AdaBoost	88.21	76.42	92.14	76.36	76.25	84.75	84.28	82.63
RF	94.65	89.34	96.43	89.93	89.04	93.18	92.88	92.21
MLP	96.08	92.18	97.39	92.32	91.98	94.94	94.78	94.24
DT	94.06	88.15	96.04	88.63	87.81	92.43	92.10	91.32
LR	91.92	83.89	94.61	84.11	83.16	89.87	89.25	88.11
SVM	92.28	84.60	94.85	85.96	84.12	90.29	89.72	88.83
KNN	91.93	83.89	94.62	83.83	83.74	89.51	89.25	88.11
Top-2	96.08	92.18	97.39	92.32	91.98	94.94	94.78	94.24
Top-3	96.31	92.65	97.54	92.75	92.54	95.21	95.10	94.59
Top-4	96.25	92.54	97.50	92.65	92.42	95.13	95.02	94.50
Top-5	95.90	91.82	97.26	92.06	91.66	94.71	94.54	93.99
Top-6	96.08	92.18	97.38	92.39	92.01	94.93	94.78	94.25
Top-7	95.90	91.82	97.26	92.06	91.61	94.73	94.54	93.99
Top-8	96.02	92.06	97.34	92.30	91.86	94.88	94.70	94.17
Top-9	95.96	91.94	97.31	92.20	91.74	94.80	94.62	94.08
Top-10	95.96	91.94	97.30	92.13	91.74	94.80	94.62	94.07
